# Protein- and Carbohydrate-Rich Supplements in Feeding Adult Black Soldier Flies (*Hermetia illucens*) Affect Life History Traits and Egg Productivity

**DOI:** 10.3390/life13020355

**Published:** 2023-01-28

**Authors:** Patrick Klüber, Emna Arous, Holger Zorn, Martin Rühl

**Affiliations:** 1 Branch for Bioresources, Fraunhofer Institute for Molecular Biology and Applied Ecology (IME), 35392 Giessen, Germany; 2 Institute of Food Chemistry and Food Biotechnology, Justus Liebig University, 35392 Giessen, Germany

**Keywords:** black soldier fly, insect rearing, rearing method, oviposition, reproduction, mating, feeding behavior, life history traits, longevity

## Abstract

The black soldier fly, *Hermetia illucens* (BSF; Diptera: Stratiomyidae), has come into the focus of research over the past decade since its larvae are polyphagous feeders with an exceptional substrate range, making them a promising candidate for the bioconversion of various organic side streams into valuable insect protein. While larval nutritional requirements have been studied in detail, basic information on adult feeding is still lacking. The reproduction of adult flies is a bottleneck and key determinant in rearing BSF, which has extensive potential for improvement. In the present study, we examined the impact of different carbohydrate (honey and d-glucose) and protein sources (*Spirulina* and *Chlorella* powder) on a variety of life history traits using a highly standardized single pair approach. Feeding a 5% honey solution was shown to make females live 2.8 d longer, become more fecund (9 egg clutches per 10 females), lay more eggs (increasing 1.7-fold to 182.4 mg per 10 females), reduce the number of failed oviposition events 3-fold and increase multiple oviposition events from 2 to 15. Additionally, female longevity after oviposition improved 1.7-fold from 6.7 to 11.5 d. In order to further optimize adult feeding, mixtures of proteins and carbohydrates with varying ratios should be tested.

## 1. Introduction

According to estimations by the United Nations (UN), the world population will reach between 9.4 and 10.2 billion by 2050 [1]. Driven by global population growth and other socio-economic factors, such as educational level, income and increasing urbanization, not only the amount of food required will change but also consumption patterns [2]. In particular, the demand for animal-derived protein is projected to increase by 70% by 2050, which has far-reaching environmental impacts on land use, water consumption, and the emission of climate-relevant greenhouse gases [3]. As a result, it is be conceivable that natural ecosystems could be converted into agricultural land, with ecosystem services being lost and biodiversity threatened worldwide. However, due to sustainability or health aspects, consumer behavior is beginning to change, and the demand for and acceptance of meat substitutes is steadily increasing. Although the alternative protein sources segment is currently less than 5% compared to the conventional meat and poultry market, its average growth rate is twice as high [4]. Some of the emerging and most promising non-meat sources include plant-derived proteins, seaweed and microalgae, fungal mycelium, and insects.

The black soldier fly, *Hermetia illucens* (BSF; Diptera: Stratiomyidae), comes with great promises for industrial purposes since its polyphagous larvae feed on a broad spectrum of organic substrates of animal and plant origins, including waste and side streams [5]. In view of the resource limitations of the food and feed producing industry, it makes sense to use BSF larvae as a bioconversion agent to upcycle side streams into high-quality insect biomass in order to conceptualize the entire production chain more efficiently and sustainably. While larval nutritional requirements and feed composition are well investigated, the number of studies focusing on feeding adults is still limited [6,7,8,9]. The reason for this is that it was previously supposed that adult flies do not feed [10] and that they fully rely on the fats they have already stored during their larval stages [11]. Despite many assertions to the contrary, recent studies have shown that the adults have a functional digestive system and that food administration affects the fly’s longevity [8,12,13]. In this context, the quality and availability of feed during larval development—as well as providing BSF adults with water and a sugar or a protein source (milk powder, peptone)—can significantly enhance egg production and extend the oviposition period and adult longevity [7,14,15].

The reproduction of the adult BSF still represents one of the largest bottlenecks in industrial mass rearing. Since it is subject to a complex regulatory network of various biotic and abiotic factors, nutritional requirements are not the only aspect that might affect egg productivity [8,9,16]. In particular, volumetric factors, such as cage size and fly density, are supposed to play a critical role in egg productivity [17,18]. BSF can be cultivated successfully in small-scale indoor systems (27 × 27 × 27 cm) in addition to conventional procedures (>100 × 100 × 100 cm) [14]. Concurrently, Park et al. showed that egg numbers and total egg weight increased at higher population densities (4, 8, and 10 kg prepupae per cage) independently of cage size [17]. Moreover, fecundity is influenced by the ambient temperature. While a female deposited 475–516 eggs at 30 °C, egg clutch size decreased to ~300 eggs at 25 °C [19]. The effects of relative humidity on fecundity of several arthropods have been investigated in detail, whereas it is still unclear whether mating and oviposition of the BSF are affected [20]. Oviposition in BSF was shown to be mediated by bacteria in a conspecific manner [21]. In particular, mating behavior and related reproductive outcome are light-dependent and most efficient when the adults are exposed to direct sunlight [10,22]. Various lighting systems of different construction, including fluorescence lamps, quartz iodine lamps, metal halide lamps, and light-emitting diodes, have already been examined for the artificial illumination of BSF adults [15,23,24,25].

The aim of the present work was to examine the impact of different carbohydrate (honey and pure d-glucose) and protein sources (*Spirulina* and *Chlorella* powder) on a variety of reproductive and lifetime parameters of BSF adults in order to evaluate their suitability for future applications. Although microalgal proteins require a higher economic effort than conventional vegetable protein sources, they are a promising and sustainable dietary supplement. For this purpose, BSF adults were sexed, separated in pairs, and fed with four concentrations of each carbohydrate or protein source, allowing us to obtain data from single pairs. At the same time, we established a simple, highly standardized method from which conclusions about the behavior of BSF adults after they have been subjected to any treatment can be drawn.

## 2. Materials and Methods

The experiments were conducted between January and June 2022 at the Fraunhofer Institute for Molecular Biology and Applied Ecology (Giessen, Germany) and the Institute of Food Chemistry and Food Biotechnology (University of Giessen, Germany).

### 2.1. Insect Rearing

BSF larvae were originally obtained from Bio.S Biogas (Grimma, Germany) in July 2018. Since then, the population has been genetically isolated (~30 generations) from others and reared continuously on chicken feed (GoldDott Eierglück, DERBY Spezialfutter, Muenster, Germany), which is frequently used as a high-quality diet [26]. The larvae were maintained in 19.5 × 16.5 × 9.5 cm (l × w × h) polypropylene boxes with 150 mg eggs (~6000 eggs) per box at 27 ± 1 °C and 65 ± 5% relative humidity in constant darkness [27,28,29]. Once ≥50% of the colony had reached the sixth instar, dark-colored prepupae were separated from the substrate using a vibratory sieve shaker (AS 200, Retsch, Haan, Germany). For pupation and subsequent metamorphosis, prepupae were transferred to mesh cages (Bioform, Nuremberg, Germany), each with a size of 60 × 60 × 90 cm (l × w × h), located in the greenhouse.

### 2.2. Feeding BSF Adults: Experimental Design and Influence on Life History Traits

About 8–10 d after pupation, adult flies emerged. Only pupae weighing 150–180 mg were selected for the experiments. To avoid injuries, adults were collected individually by using spring steel tweezers. Males and females were sexed according to external dimorphisms of the antennae and abdominal genitalia [30]. Individuals who had physiological abnormalities or who died within the first 2 d after emergence were excluded from the study.

In general, five different diet groups were established: Group 1 includes differently treated types of water (distilled water, tap water, 0.5% NaCl (*w*/*v*)-supplemented water) and no water (hereafter referred to as starvation), which were used to determine the base values of all parameters for groups 2–5. The tap water contained the following minerals: 8.23 mg/L Na^+^, 7.35 mg/L Mg^2+^, 2.07 mg/L K^+^, 34.3 mg/L Ca^2+^, 13.3 mg/L Cl^–^, <0.15 mg/L F^–^, 14.7 mg/L SO_4_^2–^, <0.02 mg/L Al, <0.1 mg/L P, 7.1 mg/L Si. The data from the tap water treatment were used as a comparison for all subsequent groups.

Group 2 (organic forest honey) and Group 3 (d-Glucose) were given carbohydrate-rich diets. Both carbohydrate supplements were diluted at 30 °C in distilled water at four concentrations (5%, 10%, 25%, and 50% (*w*/*v*)). Group 4 (*Spirulina* powder) and Group 5 (*Chlorella* powder) comprise protein-rich diets containing cyanobacterial or microalgal biomass, respectively. In contrast to the carbohydrate-rich diets, preliminary feeding experiments with both powders revealed a high lethality when supplemented with 5%. Consequently, both protein-rich supplements were diluted in distilled water at four lower concentrations (0.05%, 0.10%, 0.25%, and 0.50% (*w*/*v*)). The analytical composition and detailed information of all supplements is summarized in Table 1.

Reproductive parameters of each diet group were evaluated in 30 temporally synchronized pairs of adult flies (within 24 h of emergence) kept in a greenhouse chamber at 26 ± 1 °C, 60 ± 5% relative humidity, and with a 12 h photoperiod. Each pair was placed in its own conical 8.4 × 8.4 × 11.4 cm (l × w × h) polypropylene box, which contained a 4 × 4 cm wooden board that served as simple oviposition site, as previously described [31]. The lid was fitted with a circular 4.5 cm mesh insert, enabling gas exchange and feeding. Feeding was performed daily through spraying 2 mL of each of the corresponding prepared solutions, meticulously targeting the sides of the boxes, in a way that avoids harming the insects with the spraying pressure. Since the mating behavior of the BSF is light-dependent, all boxes were placed directly under a SON-K 400 high-pressure sodium-vapor lamp (DH Licht, Wülfrath, Germany) with the following illumination specifications: 465 W power, 56,500 lm luminous flux, 725 µmol∙s^−1^ photon flux, and 2000 K light color temperature. The light intensity was set to 300 μmol·m^−2^·s^−1^.

The sex-specific longevity of adults and the deposition of eggs were checked and documented daily. Fresh egg clutches were collected and subsequently weighed using an AT261 DeltaRange analytical balance (Mettler, Giessen, Germany). The preoviposition period was defined as the period from the adult’s emergence to the first oviposition. The oviposition period represents the time from the first to the last oviposition event within a replicate. The oviposition peak describes the day after adult emergence on which most egg clutches were deposited. For replicates without a clear peak, the mean was used instead. The adult longevity 50 (AL50) value was calculated as the period from adult emergence to ≥50% of population death. Multiple oviposition events were detected if a female deposited a second egg clutch ≥24 h after the initial oviposition. Females laying eggs were not disturbed. When calculating female longevity after oviposition, the initial oviposition date was taken as starting point if multiple oviposition events were observed. Fecundity was defined as the number of egg clutches per 10 females. Only in this context were single and multiple oviposition events expressed as one clutch to show how many females per replicate were able to deposit eggs. When determining egg clutch weight when multiple oviposition events were observed, egg mass was summed. The egg clutch size (number of eggs per clutch) was calculated assuming that an egg from a fly grown on chicken feed weighs 0.024 mg [31].

### 2.3. Data Processing and Statistics

Statistical analysis and data visualization were carried out using OriginPro 2020 (OriginLab, Northampton, MA, USA). Lifetime data including sex-specific adult longevity, total adult longevity, and female longevity after oviposition were analyzed using the non-parametric Kaplan–Meier estimation. The calculated *S(t)* survival functions were then compared pairwise using log-rank tests at an error level of α = 0.05 for statistical significance. The homogeneity of variance was assessed with the Levene’s test. Parameters within a diet group were compared by one-way analysis of variance (ANOVA) and means were separated using the Bonferroni–Holm post hoc test [32]. The linear relationship between variables was determined using Pearson product–moment correlation [33]. Diet-related effects between two data sets from different diet groups were determined using Student’s (homogeneous variance) or Welch’s (inhomogeneous variance) *t*-test and an error level of α = 0.05 for statistical significance.

## 3. Results

### 3.1. Providing the Adult BSF Different Types of Water

First, how access to different types of water affects the flies in a single-pair experimental approach was examined in order to serve as a reference for carbohydrate- and protein-rich solutions. The adult preoviposition period across treatments ranged from 7.9 to 9.1 d (Table 2) and did not differ significantly (F_2,47_ = 1.36; *p* = 0.27). The first egg clutch was found in the tap water group 6 d after emergence, followed by the distilled water and 0.5% NaCl groups after 7 d. Since no mating or egg deposition events were observed within the starvation group, all life history traits that are related to oviposition were excluded from the calculation. No significant differences were determined for the oviposition period of pairs that had access to tap water, distilled water, or 0.5% NaCl solution (F_2,6_ = 2.82; *p* = 0.14), but the mean values ranged between 1.7 and 5.0 d. In those that laid eggs, oviposition peaked at 7.8–9.0 d postemergence, with no significant difference across the treatments (F_2,6_ = 0.88; *p* = 0.46). The provision of tap water resulted in the longest oviposition span with 9 d (days 6–14), followed by distilled water with 7 d (days 7–13) and 0.5% NaCl solution with 4 d (days 7–10). Females that had access to tap water or distilled water laid 86.7% and 89.6% of the egg mass within a period of 4 d (days 7–10), respectively.

**Figure 1:**
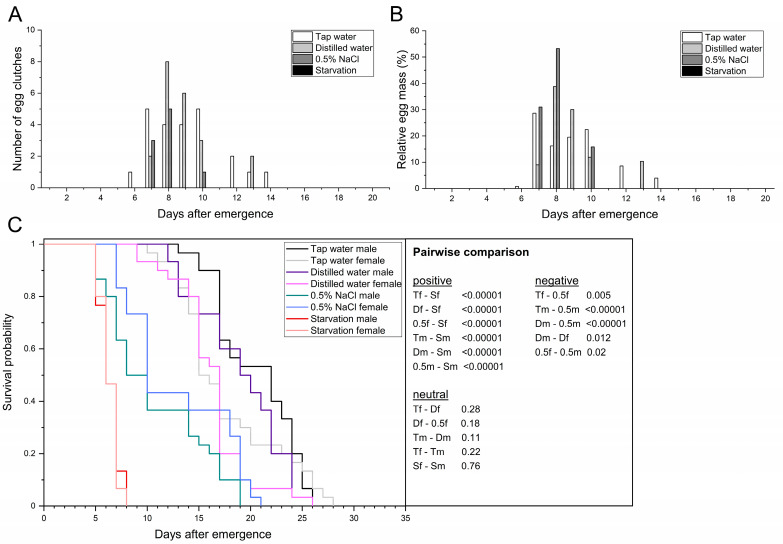
Providing adult BSF with different types of water or no water (starvation) affects oviposition and adult longevity. Temporal course of oviposition based on the absolute number of egg clutches (**A**) and the relative egg mass (**B**) collected. (**C**) Sex-dependent Kaplan–Meier survival functions of 30 pairs per treatment. Survivorship was monitored daily. *p*-values represent treatments compared pairwise via log-rank test (T = tap water; D = distilled water; 0.5 = 0.5% NaCl solution; S = starvation; f = female; m = male).

Although not significantly different, females provided with 0.5% NaCl solution laid 84.2% of the egg mass within 2 d (days 7–8), with 53.2% of the eggs being laid on day 8 (Figure 1A,B). The fecundity (number of egg clutches per ten females) ranged from 7.0 clutches in adults provided tap water to 0 clutches in adults without access to water. No significant differences were determined for the fecundity of adults provided with tap water versus distilled water (F_3,8_ = 39.60; *p* = 0.67). In contrast, provision of 0.5% NaCl solution led to a ~2.3-fold reduction in fecundity compared to both aforementioned treatments (F_3,8_ = 39.60; *p* ≤ 0.001). The same was also observed for the mean egg yield. While the tap water and distilled water treatments both resulted in more than 100 mg eggs per ten females (F_3,8_ = 19.96; *p* = 0.86), the provision of 0.5% NaCl solution significantly reduced the egg yield ~2.7-fold to 38.5 mg (F_3,8_ = 19.96; *p* ≤ 0.004). Starvation was the only treatment where no egg clutches were found (Table 2, Figure 1A,B). The total egg yield ranged from 320.8 (tap water) to 115.4 mg eggs in the 0.5% NaCl treatment. The mean egg clutch weight did not differ significantly (F_2,6_ = 1.56; *p* = 0.29) and ranged from 12.8 to 15.6 mg, with individual clutches weighing at least 7.9 and at most 31.8 mg. In addition, the mean egg clutch size did not differ significantly (F_2,6_ = 1.55; *p* < 0.29) and ranged from 651.4 to 534.3 eggs per clutch, with individual clutches comprising at least 329 and at most 1325 eggs.

Multiple ovipositions occurred in 1–2 pairs in adults provided with distilled water or tap water, whereas multiple ovipositions were not observed in the 0.5% NaCl and starvation treatments (Figure 1A,B). The second oviposition events in both treatments were observed 1 d after initial egg deposition of the corresponding female. While 9–10 pairs (≤33.3%) did not deposit eggs in the tap water and distilled water treatments, the number of pairs without oviposition increased by ≥ 110% to a total of 21 (70%) in the 0.5% NaCl treatment.

Total adult longevity of both sexes differed significantly between tap water and the other three treatments (*χ*^2^ = 260.42; *p* ≤ 0.03). Females provided with distilled water or tap water lived 16.2–17.6 d (*χ*^2^ = 1.17; *p* = 0.28), whereas males lived 19.0–20.5 d (*χ*^2^ = 2.62; *p* = 0.11, Figure 1C). After 19 d, ≥ 50% of the individuals treated with tap water were dead (AL50-value), which was not significantly different from distilled water (F_3,8_ = 19.81; *p* = 0.17). The 0.5% NaCl and starvation groups, however, led to a significant 1.6-fold or 3.0-fold reduction, respectively (F_3,8_ = 19.81; *p* ≤ 0.003). In general, adults provided any type of water lived 10.7–20.5 d and had a significantly higher survival probability compared to those not provided with water (6.3–6.4 d; *χ*^2^ = 260.42; *p* < 0.00001). Individual female longevity ranged from 5 (starvation) to 28 d when provided with tap water. On the other hand, individual males lived for at least 5 d (starvation, 0.5% NaCl) and at most 26 d when provided distilled water or tap water. Both sexes lived significantly longer when treated with tap water compared to 0.5% NaCl, with males (*χ*^2^ = 39.28; *p* < 0.00001) showing a 1.9-fold and females (*χ*^2^ = 7.83; *p* = 0.005) a 1.4-fold reduction in longevity (Figure 1C). Based on the collected data, tap water treatment was chosen as a suitable reference for the comparison with solutions rich in carbohydrates or proteins.

### 3.2. Feeding the Adult BSF Carbohydrate-Rich Solutions

Carbohydrate-rich solutions were tested over a wide range of concentrations (5–50%) for their suitability for feeding adult BSF.

#### 3.2.1. Organic Honey

The adult preoviposition period across treatments ranged from 8 to 9.1 d (Table 3) and did not differ significantly (F_4,102_ = 0.76; *p* = 0.55). All honey treatments allowed adults to mate and deposit at least 12 or more egg clutches. The first egg clutch was found in the 10% treatment 5 d after emergence, followed by the 5%, 25%, and tap water treatment after 6 d and the 50% treatment after 7 d. On one hand, no significant differences were determined for the oviposition period of pairs that had access to tap water or different concentrations of honey (F_4,10_ = 2.56; *p* = 0.10), but the mean values ranged between 1.7 and 8 d. On the other hand, the length of the oviposition period was negatively correlated with increasing honey concentration, especially from ≥ 25% (*r* = −0.69; *p* = 0.01).

**Figure 2:**
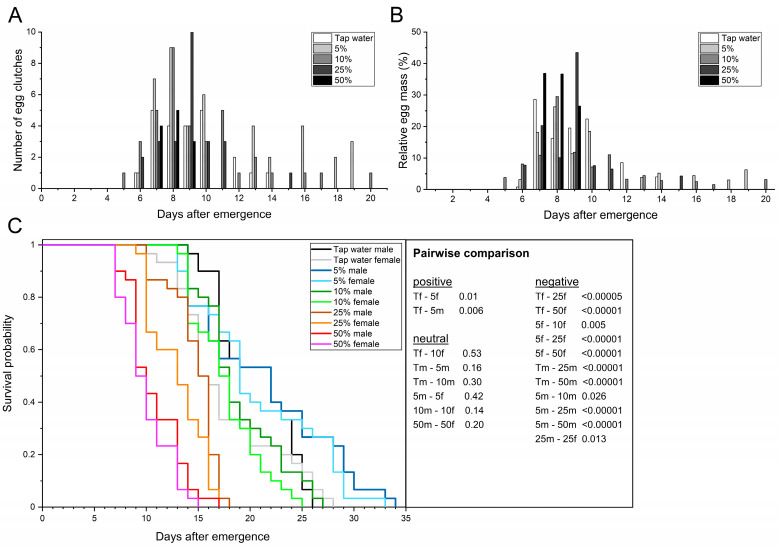
Providing adult BSF with carbohydrate-rich honey solutions (5–50% *w*/*v*). Tap water served as reference. Temporal course of oviposition based on the absolute number of egg clutches (**A**) and the relative egg mass (**B**) collected. (**C**) Sex-dependent Kaplan–Meier survival functions of 30 pairs per treatment. Survivorship was monitored daily. *p*-values represent treatments compared pairwise via log-rank test (T = tap water; 5, 10, 25, 50 = concentration of honey; f = female; m = male).

For those that laid eggs, oviposition peaked at 7.3–8.7 d postemergence, with no significant difference across the treatments (F_4,10_ = 0.72; *p* = 0.60). The provision of 10% honey resulted in the longest oviposition span of 16 d (days 5–20), followed by 5% with 14 d (days 6–19), and 25% with 10 d (days 6–15). At 3 d, only the 50% treatment resulted in a noticeably shorter oviposition span than tap water (Table 3, Figure 2A,B). Females that had access to 5% honey laid 74.2% of the egg mass within a period of 4 d (days 7–10), whereas females treated with tap water laid 86.7% within the same period. Females provided with 10% or 25% honey laid 78.5% and 95.7% of the egg mass within 6 d (days 6–11), respectively. The 50% treatment resulted in the females laying the entire egg mass in 3 d (days 7–9, Figure 2B). The fecundity ranged from 4 clutches in adults provided 50% honey to 9.3 clutches in adults with access to 10% honey. No significant differences were determined for the fecundity of adults provided with tap water and 5–25% honey treatments, while access to 50% honey led to a significant 1.8-fold reduction in fecundity compared to tap water (F_4,10_ = 15.11; *p* = 0.003).

In general, the fecundity strongly negatively correlated with increasing honey concentrations (*r* = −0.92; *p* = 0.00002), indicating that lower concentrations (5–10%) are more suitable for the flies. The same was also observed for the mean egg yield. With 182.4 mg eggs per ten females, the 5% treatment significantly increased the mean egg yield by 70.6% compared to tap water (F_4,10_ = 12.13; *p* = 0.006), whereas the 10–50% treatments did not differ significantly (F_4,10_ = 12.13; *p* ≤ 0.58). Although not significantly different from tap water, increasing honey concentrations reduced the mean egg yield significantly by up to 71.2% (52.5 mg) compared to 5% honey (*r* = −0.91; *p* = 0.00005). The total egg yield ranged from 157.6 (50% honey) to 489.5 mg eggs in the 5% honey treatment. The mean egg clutch weight did not differ significantly (F_4,10_ = 2.65; *p* < 0.1) and ranged from 12.7 to 20.3 mg, with individual clutches weighing at least 3.4 and at most 42.0 mg. In addition, the mean egg clutch size did not differ significantly (F_4,10_ = 2.64; *p* = 0.09) and ranged from 527.8 to 847.4 eggs per clutch, with individual clutches comprising at least 163 and at most 1750 eggs. Both mean egg clutch size and mean egg clutch weight correlated negatively with increasing honey concentration (*r* = −0.70; *p* = 0.01).

With 9–15 pairs, multiple oviposition events occurred noticeably more often in adults provided honey concentrations between 5 and 10%, whereas only 1–2 multiple ovipositions were observed in the 50% and tap water treatments (Table 3, Figure 2A,B). Interestingly, the second oviposition of females of the 10 and 5% treatments occurred 4.1 d or 5.5 d after initial oviposition (both ranged between 1–11 d), respectively. When comparing the initial and second oviposition, it was noticeable that 41.9 (83.3 mg; 10% honey) and 32.4% (95.8 mg; 5% honey) of the egg mass of the corresponding females was laid during the second oviposition. In this context, the egg mass of the second oviposition accounted for 17.1–19.6% of the total egg yield, whereas it was 11.5% (36.9 mg) for females treated with tap water. While 2–3 pairs (≤10%) did not deposit eggs in the 10 and 5% treatments, the number of pairs without oviposition increased ≥2.7-fold to a total of 11 (36.7%) in the 25% treatment and ≥5-fold to a total of 18 (60%) in the 50% treatment.

Total adult longevity of the flies that had access to tap water and 10% honey was the same but differed significantly from the other treatments (*χ*^2^ = 299.97; *p* ≤ 0.005). Females provided with tap water or 5% honey lived 17.6–20.8 d (*χ*^2^ = 6.36; *p* = 0.01), whereas males lived 20.5–21.4 d (*χ*^2^ = 2.00; *p* ≤ 0.16). After 17.7 and 20 d, respectively, ≥50% of the individuals treated with 5% and 10% honey were dead, which was not significantly different from tap water (F_4,10_ = 17.57; *p* ≥ 0.38). However, the 25 and 50% treatment led to a significant 1.3–2.0-fold reduction (F_4,10_ = 17.57; *p* < 0.01). The provision of 5% honey resulted in the highest longevity regardless of the sex (Table 3, Figure 2C). The sex-specific longevity of males and females treated with 25 (*χ*^2^ = 21.85; *p* < 0.00001 and *χ*^2^ = 16.51; *p* < 0.00005) or 50% honey (*χ*^2^ = 54.27; *p* < 0.00001 and *χ*^2^ = 47.51; *p* < 0.00001) was significantly reduced compared to tap water and both lower honey concentrations. In particular, comparing the 5 and 50% honey treatments, longevity was reduced 2.0–2.1-fold in males and females. Individual female longevity ranged from 7 (50% honey) to 33 d when provided 5% honey (Figure 2C). On the other hand, individual males lived for at least 7 (50% honey) and at most 34 d when provided 5% honey. In general, total adult longevity (*r* = −0.94; *p* < 0.00001) as well as male (*r* = −0.96; *p* < 0.00001) and female (*r* = −0.94; *p* < 0.00001) longevity were strongly negatively correlated with increasing honey concentration, indicating that the 5% concentration is most suitable for the flies.

#### 3.2.2. d-Glucose

The adult preoviposition period across treatments ranged from 6.1 to 9.1 d (Table 4) and was moderately negatively correlated with increasing glucose concentration, especially from ≥ 25% glucose (*r* = −0.41; *p* = 0.0004). Adults that had access to 25 and 50% glucose showed a significantly shorter preoviposition period than those provided tap water (F_4,64_ = 5.84; *p* ≤ 0.0005). All glucose treatments allowed adults to mate and deposit at least eight or more egg clutches. The first egg clutches were found 5 d after emergence in all glucose treatments, followed by tap water 1 d later.

**Figure 3:**
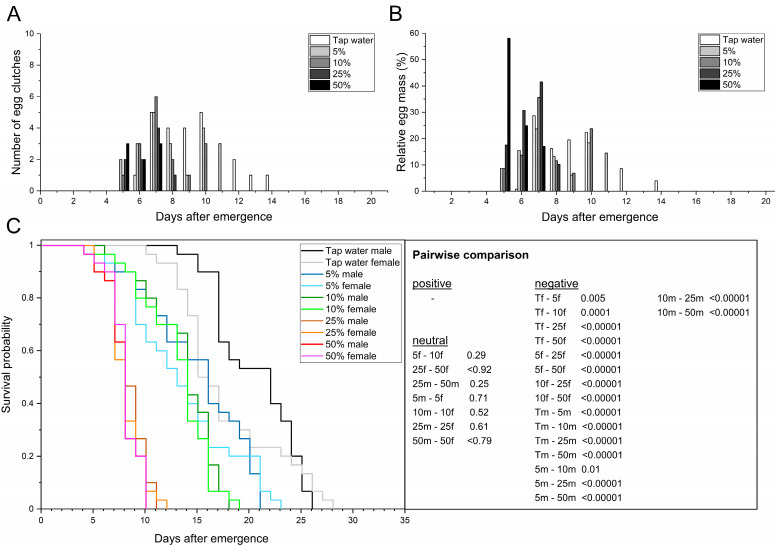
Providing adult BSF with glucose solutions (5–50% *w*/*v*). Tap water served as reference. Temporal course of oviposition based on the absolute number of egg clutches (**A**) and the relative egg mass (**B**) collected. (**C**) Sex-dependent Kaplan–Meier survival functions of 30 pairs per treatment. Survivorship was monitored daily. *p*-values represent treatments compared pairwise via log-rank test (T = tap water; 5, 10, 25, 50 = concentration of glucose; f = female; m = male).

No significant differences or correlations were determined for the oviposition period of pairs provided tap water or different concentrations of glucose (F_4,10_ = 2.62; *p* = 0.10), but the mean values ranged between 1.7 and 5 d. For those that laid eggs, oviposition peaked at 5.8–8.3 d postemergence, with no significant difference across the treatments (F_4,10_ = 2.50; *p* = 0.11). In general, all glucose treatments resulted in a shorter oviposition span than tap water (Table 4, Figure 3A,B). Although not significant, there was a noticeable shortening of the oviposition span with increasing glucose concentration, decreasing from 7 (days 5–11) to 3 d (days 5–7). Females that had access to 5% or 10% glucose laid ≥ 91.3% of the egg mass within a period of 6 (days 6–11) and 5 d (days 6–10), whereas females provided with 25% glucose laid 100% within 4 d (days 5–8). The 50% treatment resulted in the females laying the entire egg mass in 3 d (days 5–7, Figure 3B).

The fecundity ranged from 2.7 clutches in adults provided with 50% glucose to 5.7 clutches in adults with access to 5% glucose. No significant differences were determined for the fecundity of adults provided with tap water and 5% glucose, while 10–50% glucose treatments led to a significant 1.5–2.6-fold reduction in fecundity compared to tap water (F_4,10_ = 16.5; *p* < 0.004). In general, the fecundity was strongly negatively correlated with increasing glucose concentration (*r* = −0.81; *p* = 0.001). The same was also observed for the mean egg yield (*r* = −0.79; *p* = 0.002). With ≤ 38.2 mg eggs per ten females, the 25 and 50% treatments significantly reduced the mean egg yield by up to 69.8% compared to tap water (F_4,10_ = 7.56; *p* < 0.002), whereas the 5–10% treatments did not differ significantly (F_4,10_ = 7.56; *p* ≤ 0.13). The total egg yield ranged from 96.9 (50% glucose) to a maximum of 241.0 mg eggs in the 5% treatment, representing a ≥ 24.9% reduction compared to tap water. The mean egg clutch weight did not differ significantly (F_4,10_ = 1.74; *p* = 0.22) and ranged from 11.8 to 15.2 mg, with individual clutches weighing at least 3.9 and at most 22.5 mg. In addition, the mean egg clutch size did not differ significantly (F_4,10_ = 1.75; *p* = 0.22) and ranged from 490.0 to 633.8 eggs per clutch, with individual clutches comprising at least 163 and at most 938 eggs. Both mean egg clutch size and mean egg clutch weight were negatively correlated with increasing glucose concentration (*r* = −0.57; *p* = 0.05).

With four pairs, multiple oviposition events occurred most frequently in adults provided with 5% glucose, whereas two multiple ovipositions were observed in the 10% and tap water treatments (Table 4, Figure 3A,B). No multiple ovipositions were found in the 25 and 50% treatments. The second oviposition in females of the 10 and 5% treatments occurred 1 (both within 1 d) or 1.8 d (1–2 d) after initial oviposition. When comparing the initial and second oviposition, it was noticeable that 24.9 (8.5 mg; 10% glucose) and 32.7% (24.5 mg; 5% glucose) of the egg mass of the corresponding females was laid during the second oviposition. The egg mass of the second oviposition accounted for 4.0–10.2% of the total egg yield. All glucose treatments resulted in a higher number of pairs that did not deposit eggs compared to those provided tap water. While 13 pairs (43.3%) did not deposit eggs in the 5% treatment, the number of pairs without oviposition increased 1.6-fold to a total of 21 (70%) in the 25% treatment and 1.7-fold to a total of 22 (73.3%) in the 50% treatment. The number of pairs without oviposition was positively correlated with increasing glucose concentration (*r* = 0.81; *p* = 0.001).

Total adult longevity (*χ*^2^ = 276.52; *p* < 0.00001), as well as female and male longevity (*χ*^2^ = 279.39; *p* < 0.00001), differed significantly across the treatments. Females (*χ*^2^ = 7.76; *p* ≤ 0.005) and males (*χ*^2^ = 21.70; *p* < 0.00001) that had access to tap water lived significantly longer compared to those treated with any glucose concentration. The same was also observed for the AL50 values. While ≥ 50% of the individuals treated with tap water were dead after 19 d, provision of glucose led to a significant 1.4–2.5-fold reduction (F_4,10_ = 18.99; *p* = 0.0001). Within the glucose treatments, administration of 5% glucose led to the highest longevity regardless of sex (Table 4, Figure 3C). The sex-specific longevity of males and females treated with 25 (*χ*^2^ =37.89; *p* < 0.00001 and *χ*^2^ = 30.99; *p* < 0.00001) or 50% glucose (*χ*^2^ = 39.78; *p* < 0.00001 and *χ*^2^ = 31.22; *p* < 0.00001) was significantly reduced compared to both lower glucose concentrations. In particular, comparing the 5 and 50% treatments, longevity was reduced 1.9-fold in males and 1.7-fold in females. Individual female longevity ranged from 4 (50% glucose) to 23 d when provided 5% glucose, while individual males lived for at least 4 and at most 21 d (Figure 3C). In general, total adult longevity (*r* = −0.85; *p* < 0.0004) as well as male (*r* = −0.87; *p* < 0.0002) and female (*r* = −0.82; *p* < 0.001) longevity were strongly negatively correlated with increasing glucose concentration.

### 3.3. Feeding the Adult BSF Protein-Rich Solutions

In contrast to the carbohydrate-rich diets, preliminary feeding experiments revealed a high lethality when supplemented with 5% (data not shown). Consequently, protein-rich solutions were tested for their suitability for feeding adult BSF over a range of 0.05–0.5%.

#### 3.3.1. *Spirulina* Powder

The adult preoviposition period across treatments ranged from 5.7 to 9.1 d and was negatively correlated with increasing *Spirulina* concentration, especially from ≥ 0.25% (*r* = −0.75; *p* = 0.005). Adults treated with 0.25 and 0.5% *Spirulina* showed a significantly shorter preoviposition period than those provided tap water (F_4,90_ = 6.72; *p* ≤ 0.0001). All *Spirulina* treatments allowed adults to mate and deposit at least 15 or more egg clutches. The first egg clutches were found 4 d after emergence in the 0.05, 0.25, and 0.5% treatments, followed by 0.1% *Spirulina* and tap water 2 d later (Table 5). The oviposition period ranged between 1.7–8.7 d, with the period of adults provided with 0.05% *Spirulina* being significantly longer than those provided with higher concentrations or tap water (F_4,10_ = 13.65; *p* ≤ 0.005). Additionally, the length of the oviposition period was negatively correlated with increasing *Spirulina* concentration (*r* = −0.69; *p* = 0.01). For those that laid eggs, oviposition peaked at 5.7–8.3 d postemergence, with no significant difference across the treatments (F_4,10_ = 3.18; *p* = 0.06).

**Figure 4:**
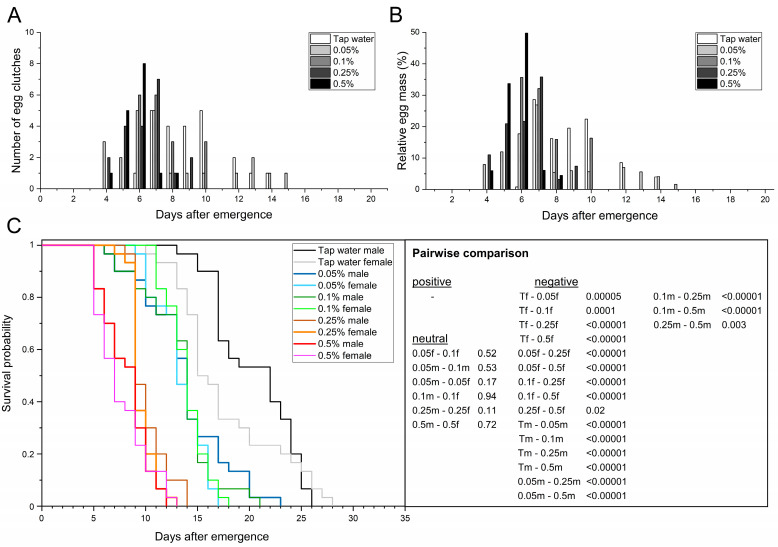
Providing adult BSF with protein-rich *Spirulina* solutions (0.05–0.5% *w*/*v*). Tap water served as reference. Temporal course of oviposition based on the absolute number of egg clutches (**A**) and the relative egg mass (**B**) collected. (**C**) Sex-dependent Kaplan–Meier survival functions of 30 pairs per treatment. Survivorship was monitored daily. *p*-values represent treatments compared pairwise via log-rank test (T = tap water; 0.05, 0.1, 0.25, 0.5 = concentration of *Spirulina*; f = female; m = male).

At 12 d (days 4–15), pairs treated with 0.05% *Spirulina* had the longest oviposition span, followed by tap water with 9 d (days 6–14) and the other *Spirulina* treatments with 5–6 d (Table 5, Figure 4A,B). Females that had access to 0.05% *Spirulina* laid 81.6% of the egg mass within a period of 7 d (days 4–10), while females provided with 0.25% or 0.1% *Spirulina* laid 89.4% in 4 d (days 4–7) and 83.7% in 3 d (days 6–8). The 0.5% treatment resulted in the females laying 83.5% of the egg mass in 2 d (days 5–6), with 49.8% on day 6 (Figure 4B).

The fecundity ranged from 5.0 clutches in adults provided with 0.5% *Spirulina* to 7.0 clutches in adults with access to 0.05% *Spirulina*. Although the fecundity did not differ significantly across treatments (F_4,10_ = 2.03; *p* = 0.17), 0.5% *Spirulina* treatment led to a 1.4-fold reduction compared to tap water. In general, the fecundity is negatively correlated with increasing *Spirulina* concentration (*r* = −0.56; *p* = 0.03). The mean egg yield did not differ significantly across treatments (F_4,10_ = 1.57; *p* = 0.26) and was not significantly correlated with the *Spirulina* concentration (*r* = −0.48; *p* = 0.07). Nevertheless, the 0.5% treatment resulted in the lowest mean egg yield at 76.4 mg eggs per ten females, representing a 28.5% reduction compared to tap water (Table 5). The total egg yield ranged from 229.2 (0.5% *Spirulina*) to a maximum of 323.1 mg eggs in the 0.05% treatment, representing a 29.1% reduction. The mean egg clutch weight did not differ significantly (F_4,10_ = 0.11; *p* = 0.98) and ranged from 15.0 to 16.2 mg, with individual clutches weighing at least 3.8 and at most 29.2 mg. The same was also observed for the mean egg clutch size, which ranged from 623.6 to 674.7 eggs per clutch. Individual egg clutches comprised at least 158 and at most 1217 eggs.

With two pairs, multiple oviposition occurred most frequently in adults provided 0.05% *Spirulina* and tap water, whereas one multiple oviposition event was observed in the 0.5% treatment (Table 5, Figure 4A,B). No multiple ovipositions were found in the 0.1 and 0.25% treatments. The second oviposition in females of the 0.5 and 0.05% treatments occurred 1 or 4 d after initial oviposition. When comparing the initial and second oviposition, it is noticeable that 45.3 (6.5 mg; 0.5% *Spirulina*) and 48.5% (23.9 mg; 0.05% *Spirulina*) of the egg mass of the corresponding females was laid during the second oviposition. The egg mass of the second oviposition accounted for 2.8–7.4% of the total egg yield. All *Spirulina* treatments resulted in a similar or higher number of pairs that did not deposit eggs compared to those provided tap water. While nine pairs (30%) did not deposit eggs in the 0.05% and tap water treatments, the number of pairs without oviposition increased 1.3-fold to a total of 12 (40%) in the 0.1% treatment and 1.7-fold to a total of 15 (50%) in the 0.5% treatment. The number of pairs without oviposition was positively correlated with increasing *Spirulina* concentration (*r* = 0.54; *p* < 0.03).

Total adult longevity (*χ*^2^ = 312.84; *p* < 0.00001), as well as female and male longevity (*χ*^2^ = 318.21; *p* < 0.00001), differed significantly across the treatments (Table 5, Figure 4C). Females (*χ*^2^ = 14.73; *p* ≤ 0.0001) and males (*χ*^2^ = 30.11; *p* < 0.00001) that had access to tap water lived significantly longer compared to those treated with any *Spirulina* concentration. The same was also observed for the AL50 values. While ≥ 50% of the individuals treated with tap water were dead after 19 d, provision of *Spirulina* led to a significant 1.4–2.6-fold reduction (F_4,10_ = 22.08; *p* = 0.00006). Within the *Spirulina* treatments, administration of 0.05% (males) and 0.1% (females) led to the highest longevity and did not differ significantly (*χ*^2^ = 0.40; *p* ≤ 0.53). The sex-specific longevity of males and females treated with 0.25 (*χ*^2^ = 21.96; *p* < 0.00001 and *χ*^2^ = 41.01; *p* < 0.00001) or 0.5% *Spirulina* (*χ*^2^ = 38.82; *p* < 0.00001 and *χ*^2^ = 46.16; *p* < 0.00001) was significantly reduced compared to both lower *Spirulina* concentrations. In particular, comparing the 0.05 and 0.5% treatments, longevity was reduced 1.7–1.8-fold in both males and females. Individual female longevity ranged from 5 (0.5% *Spirulina*) to 18 d when provided with 0.1% *Spirulina*, while individual males lived for at least 5 (0.5% *Spirulina*) and was at most 23 d (0.05% *Spirulina*, Figure 4C). In general, total adult longevity (*r* = −0.67; *p* < 0.00001) as well as male (*r* = −0.61; *p* < 0.00001) and female (*r* = −0.75; *p* < 0.00001) longevity were negatively correlated with increasing *Spirulina* concentration.

#### 3.3.2. *Chlorella* Powder

The adult preoviposition period across treatments ranged from 5.7 to 9.1 d (Table 6) and was not significantly correlated with increasing *Chlorella* concentrations (*r* = −0.42; *p* = 0.17). Adults that had access to 0.1–0.5% *Chlorella* showed a significantly shorter preoviposition period than those provided tap water (F_4,82_ = 4.48; *p* ≤ 0.002). All *Chlorella* treatments allowed adults to mate and deposit at least 10 or more egg clutches. The first egg clutches were found 3 d after emergence in the 0.05 and 0.1% treatments, followed by 0.25 and 0.5% treatments 1 d after. No significant differences or correlations were determined for the oviposition period of pairs provided with tap water or different concentrations of *Chlorella* (F_4,10_ = 2.87; *p* ≤ 0.08), but the mean values ranged between 2 and 7.7 d. For those that laid eggs, oviposition peaked at 5.0–8.3 d postemergence, with no significant difference across the treatments (F_4,10_ = 3.75; *p* ≤ 0.50).

**Figure 5:**
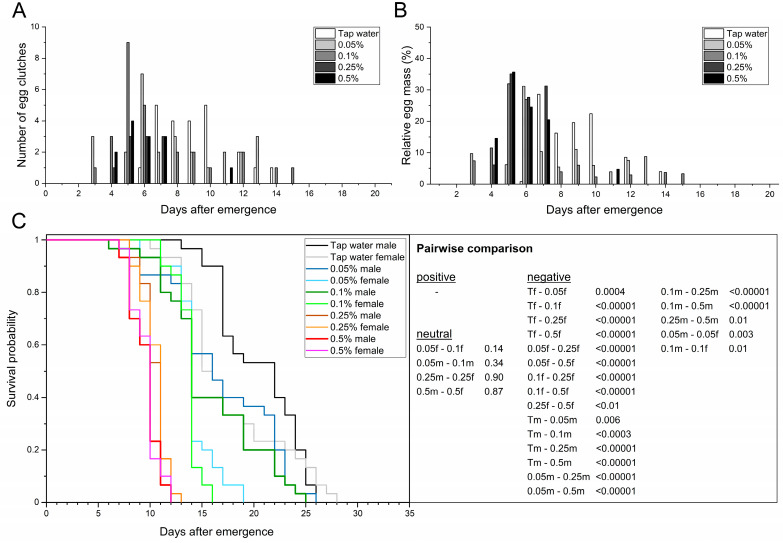
Providing adult BSF with protein-rich *Chlorella* solutions (0.05–0.5% *w*/*v*). Tap water served as reference. Temporal course of oviposition based on the absolute number of egg clutches (**A**) and the relative egg mass (**B**) collected. (**C**) Sex-dependent Kaplan–Meier survival functions of 30 pairs per treatment. Survivorship was monitored daily. *p*-values represent treatments compared pairwise via log-rank test (T = tap water; 0.05, 0.1, 0.25, 0.5 = concentration of *Chlorella*; f = female; m = male).

With 11 (days 3–13) and 13 d (days 3–15), adults provided with 0.05 and 0.1% *Chlorella* showed longer oviposition spans than those provided with tap water. In contrast, 0.25 and 0.5% treatments resulted in shorter oviposition spans of 4–8 d (Table 6, Figure 5A,B). Females that had access to 0.05 or 0.1% *Chlorella* laid ≥ 77.8% of the egg mass within a period of 8 (days 3–10) and 4 d (days 3–6), respectively, whereas females provided with 0.5% *Chlorella* laid 95.3% within 4 d (days 4–7). The 0.25% treatment resulted in the females laying the entire egg mass within 4 d (days 4–7, Figure 5B). The fecundity ranged from 3.3 clutches in adults provided with 0.25% *Chlorella* to 7.3 clutches in adults with access to 0.05–0.1% *Chlorella*. No significant differences were determined for the fecundity of adults provided with tap water and 0.05–0.1% *Chlorella*, while the 0.25–0.5% treatments led to a significant 2.1–1.8-fold reduction in fecundity compared to tap water (F_4,10_ = 9.67; *p* ≤ 0.007). In general, the fecundity was strongly negatively correlated with increasing *Chlorella* concentration (*r* = −0.72; *p* = 0.002). The same was also observed for the mean egg yield (*r* = −0.74; *p* = 0.002). With ≤ 55.8 mg eggs per ten females, the 0.25 and 0.5% treatments significantly reduced the mean egg yield by up to 52.9% compared to tap water (F_4,10_ = 9.40; *p* < 0.008), whereas the 0.05–0.1% treatments did not differ significantly (F_4,10_ = 9.40; *p* ≤ 0.89). The total egg yield ranged from 150.8 (0.5% *Chlorella*) to a maximum of 367.6 mg eggs in the 0.05% treatment, representing a maximum reduction of 53% compared to tap water. The mean egg clutch weight did not differ significantly (F_4,10_ = 0.51; *p* = 0.73) and ranged from 14.4 to 16.7 mg, with individual clutches weighing at least 5.1 and at most 27.3 mg. In addition, mean egg clutch size did not differ significantly and ranged from 600.6 to 694.3 eggs per clutch, with individual clutches comprising at least 213 and at most 1138 eggs (F_4,10_ = 0.50; *p* = 0.74). Both mean egg clutch size and mean egg clutch weight were not correlated with increasing *Chlorella* concentration (*r* = −0.20; *p* = 0.47). With five pairs, multiple oviposition events occurred most frequently in adults provided with 0.05 and 0.1% *Chlorella*, whereas one multiple oviposition was observed 1 d after initial oviposition in the 0.5% treatment. No multiple oviposition was found in the 0.25% treatment (Table 6, Figure 5A,B). The second oviposition in females of the 0.1 and 0.05% treatments occurred 2.6 (ranged between 1–6 d) or 3.2 d (ranged between 2–6 d), respectively, after initial oviposition. When comparing the initial and second oviposition, it was noticeable that 34.9 (29.7 mg; 0.1% *Chlorella*) and 48.1% (48.5 mg; 0.05% *Chlorella*) of the egg mass of the corresponding females was laid during the second oviposition. In contrast, 70.5% (7.4 mg) was laid during the second oviposition event in the 0.5% treatment. The egg mass of the second oviposition accounted for 4.4–13.2% of the total egg yield. Comparing all groups and their corresponding concentrations, supplementation with 5% honey led to the highest number of multiple oviposition events (Table 2, Table 3, Table 4, Table 5 and Table 6).

In the 0.25 and 0.5% treatments, the number of pairs that did not deposit eggs was ≥2-fold higher compared to those provided tap water. While eight pairs (26.7%) did not deposit eggs in the 0.05 and 0.1% treatments, the number of pairs without oviposition increased 2.3-fold to a total of 18 (60%) in the 0.5% treatment and 2.5-fold to a total of 20 (66.7%) in the 0.25% treatment. The number of pairs without oviposition was positively correlated with increasing *Chlorella* concentration (*r* = 0.72; *p* = 0.002).

Total adult longevity (*χ*^2^ = 309.06; *p* < 0.00001), as well as female and male longevity (*χ*^2^ = 319.28; *p* < 0.00001), differed significantly across the treatments. Females (*χ*^2^ = 12.75; *p* < 0.0004) and males (*χ*^2^ = 7.47; *p* ≤ 0.006) that had access to tap water lived significantly longer compared to those treated with any *Chlorella* concentration. The same was observed for the AL50 values. While ≥ 50% of the individuals treated with tap water were dead after 19 d, provision of *Chlorella* led to a significant 1.4–2.0-fold reduction (F_4,10_ = 15.39; *p* = 0.004). Within the *Chlorella* treatments, administration of 0.05% *Chlorella* led to the highest longevity regardless of sex (Table 6, Figure 5C). The sex-specific longevity of males and females treated with 0.25 (*χ*^2^ = 37.71; *p* < 0.00001 and *χ*^2^ = 47.13; *p* < 0.00001) or 0.5% *Chlorella* (*χ*^2^ = 42.69; *p* < 0.00001 and *χ*^2^ = 51.43; *p* < 0.00001) was significantly reduced compared to both lower *Chlorella* concentrations. In particular, comparing the 0.05 and 0.5% treatments, longevity was reduced 1.8-fold in males and 1.5-fold in females. Individual female longevity ranged from 7 (0.5% *Chlorella*) to 19 d when provided with 0.05% *Chlorella*, while individual males lived for at least 6 (0.05–0.1% *Chlorella*) and was at most 26 d (0.05% *Chlorella*, Figure 5C). In general, total adult longevity (*r* = −0.65; *p* < 0.00001) as well as male (*r* = −0.68; *p* < 0.00001) and female (*r* = −0.66; *p* < 0.00001) longevity were negatively correlated with increasing *Chlorella* concentration.

### 3.4. Female Longevity after Oviposition

In general, most female BSF die within a few hours to 4 days postoviposition, while Samayoa and colleagues reported a maximum longevity of 9 days [15,34,35]. This parameter allows an assessment of the availability of energy reserves in flies after mating and deposition of eggs and indicates to what extent adult feeding affects their metabolic maintenance.

**Figure 6:**
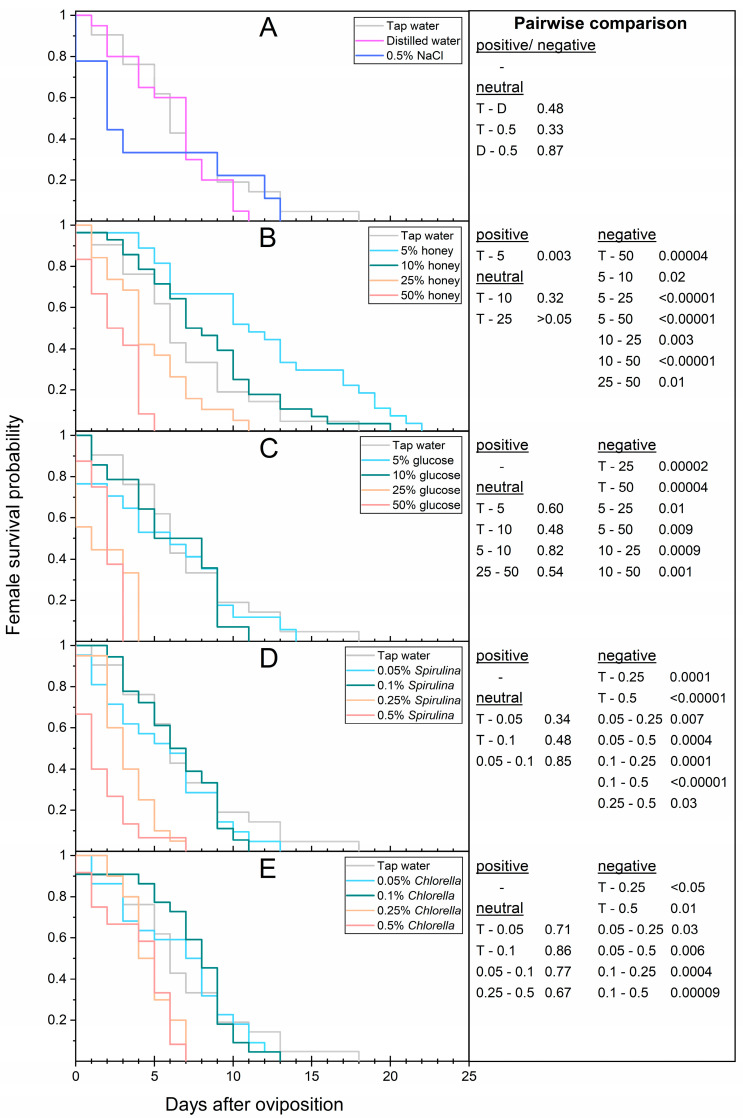
Kaplan–Meier survival functions of the female longevity after oviposition. Thirty females were fed (**A**) different types of water or no water (starvation), (**B**) honey, (**C**) glucose, (**D**) *Spirulina*, or (**E**) *Chlorella* solutions. If multiple oviposition events were observed, longevity was calculated based on the date of initial oviposition. Tap water served as reference. Survivorship was monitored daily. *p*-values represent treatments compared pairwise via log-rank test (T = tap water; D = distilled water; 5–50 = concentration of honey or glucose; 0.05–0.5 = concentration of *Spirulina* or *Chlorella*).

Since no mating or oviposition event was observed in the starvation group, the corresponding flies were excluded from the calculation. The female longevity after oviposition of flies provided with tap water, distilled water, and 0.5% NaCl solution varied between 6.7–4.8 d and did not differ significantly (*χ*^2^ = 0.91; *p* = 0.64, Table 2, Figure 6A). Two females fed with 0.5% NaCl died within 24 h after oviposition.

The longevity after oviposition of females that had access to tap water and 10–25% honey was the same and ranged between 4.8–8.4 d but differed significantly from the 5% honey treatment with 11.5 d in average and a remarkable maximum individual longevity of 22 d (*χ*^2^ = 5.88; *p* ≤ 0.02, Table 3, Figure 6B). At 2.4 d, feeding 50% honey solution led to a significant 2.8-fold and 4.8-fold reduction in longevity compared to tap water (*χ*^2^ = 17.08; *p* < 0.00004) or 5% honey (*χ*^2^ = 33.80; *p* < 0.00001), respectively. Moreover, the individual longevity ranged between 0–5 d after oviposition.

None of the glucose concentrations resulted in an improvement in female longevity after oviposition. However, with 5.6–6.1 d, the 5 and 10%-treated flies (*χ*^2^ = 0.27; *p* = 0.60 and *χ*^2^ = 0.49; *p* = 0.48) did not differ significantly from the tap water group (Table 4, Figure 6C). At 2.0–2.1 d after oviposition, feeding 25 (*χ*^2^ = 18.03; *p* = 0.00002) and 50% glucose (*χ*^2^ = 16.78; *p* = 0.00004) solution led to a significant 3.4- and 3.2-fold reduction in longevity compared to tap water. The individual longevity of both concentrations ranged from a minimum of 0 d to a maximum of 3 (50% glucose) or 4 d (25% glucose).

None of the *Spirulina* concentrations resulted in an improvement in female longevity after oviposition. However, with 5.5–6.4 d, the 0.05 and 0.1%-treated flies (*χ*^2^ = 0.92; *p* = 0.34 and *χ*^2^ = 0.49; *p* = 0.48) did not differ significantly from the tap water group (Table 5, Figure 6D). At 1.7–3.4 d after oviposition, feeding 0.5 (*χ*^2^ = 20.38; *p* < 0.00001) and 0.25% *Spirulina* (*χ*^2^ = 14.38; *p* = 0.0001) solution led to a significant 3.9- and 2.0-fold reduction in longevity compared to tap water. The individual longevity of both the 0.25 and 0.5% *Spirulina* groups ranged from a minimum of 0 d to a maximum of 7 d.

None of the *Chlorella* concentrations resulted in a significant improvement in female longevity after oviposition. However, with 6.6–7.4 d, the 0.05 and 0.1%-treated flies (*χ*^2^ = 0.14; *p* = 0.71 and *χ*^2^ = 0.03; *p* = 0.86) did not differ significantly from the tap water group (Table 6, Figure 6E). At 4.9–4.0 d after oviposition, feeding 0.25 (*χ*^2^ = 4.01; *p* < 0.05) and 0.5% *Chlorella* (*χ*^2^ = 6.05; *p* = 0.01) solution led to a significant 1.4- and 1.7-fold reduction in longevity compared to tap water. The individual longevity of both concentrations ranged from a minimum of 0 (0.5% *Chlorella*) or 2 d (0.25% *Chlorella*) to a maximum of 7 d.

## 4. Discussion

Due to its high bioconversion capacity and low nutritional demands, the BSF seems to be one of the most promising insect species for the production of alternative proteins, especially since the larvae can valorize organic waste streams. While aspects of larval nutrition, biology, and development are well known, adult flies are mostly overlooked and must be better researched in order to optimize egg production [7,36]. This comprehensive study is the first one that demonstrates how several diets and concentrations thereof affect life-history traits of adults using a highly standardized single pair approach that allowed the exact investigation of various reproductive and longevity-related parameters.

Contrary to what has long been assumed, BSF adults appear to feed rather than relying solely on the fat body built up during juvenile development [10,11,37]. In insects, the digestive system represents the meeting point of various processes like the ingestion and digestion of nutrients, metabolism, defense against both biotic and abiotic factors as well as the absorption and storage, and that is why it plays a crucial part in the life of insects. It has been shown recently that the adult BSF indeed has a functional digestive system and that feed administration affects the fly’s longevity considerably [12]. In this context, Bruno and colleagues discussed the presence of typical and suitable mouthparts that may permit the adult BSF to ingest feed. These mouthparts are covered with sensilla that permit mechano- and chemoreception. Like most dipterans, BSF did not present any mandibles or maxillae and featured a labellum that expanded from the distal part of the labium [12]. As in *Musca domestica* (Diptera: Muscidae), the adult BSF possessed prestomal tooth-like structures that could be used for semi-solid substrate scraping [38]. During metamorphosis, the larval midgut is replaced by an epithelium that remains in place until the adult stage is reached. This midgut epithelium, the activity of which is supported by endocrine and stem cells, is responsible for digestion and is composed of columnar cells [39]. As the BSF prepares to become an adult, midgut stem cells proliferate and differentiate forming the epithelium that becomes later on the midgut of the fly [40]. The midgut of the adult BSF presents three regions that are morphologically different. The presence of developed microvilli, abundant mitochondria, endoplasmic reticula and secretory vesicles, moreover the enzymatic activity, suggest the secretory and absorbing activity as well as the full functionality of the alimentary canal [12].

In accordance with our findings (Table 2), recent studies have shown that starved flies and flies that were fed with tap water exhibited a high variance in longevity. While both sexes lived less than 10 d (7.7 d males, 8.7 d females) when starved, water supplementation significantly extended adult longevity more than two-fold to 17.2 and 18 d in a single specimen approach [41]. For instance, Tomberlin et al. [42] postulated a mean longevity of 14 or 17 d for adult females and males when incubated at 27 °C and provided water, whereas Nakamura et al. [14] even reported 21 d. Interestingly, Lupi et al. could not find differences in the ovary development of females, whereas our data suggest that starved females are incapable of laying eggs [41]. With the exception of 1.5 d in total adult longevity, no significant difference was identified between the distilled water and tap water group. Altogether, these data corroborate that water plays a key role in adult fitness and performance [7,37]. The influence of salinity on the development and survival of dipteran larvae has been extensively studied, but little is known about the adult flies [43]. For example, representatives of the genus *Ephydra* (Diptera: Ephydridae) are known for their high salt tolerance [44], whereas other flies may have problems with osmoregulation even at low salt concentrations, leading to reduced longevity. In the BSF adults, the impact of the 0.5% NaCl treatment was greater than that of the distilled water treatment. Presumably, the lack of minerals in the water can be better compensated by the flies through osmoregulation than an excess of these.

However, since providing protein- or carbohydrate-rich aqueous solutions to adults has been described as promising strategy to enhance egg production and ensure a more successful industrial rearing process [7,12,41], the focus of this study was to compare both macronutrients to water (honey/d-glucose and *Spirulina*/*Chlorella* powder). BSF females demonstrated a preference towards a supplementation with honey solution compared to all the other tested treatments. A concentration of 5% honey dissolved in water was shown to make females live 2.8 d longer, become more fecund (9 egg clutches per 10 females), lay more eggs (increasing 1.7-fold to 182.4 mg per 10 females), reduce the number of failed oviposition events three-fold and at the same time improve multiple oviposition events from 2 to 15 (every second female). Additionally, the female longevity after oviposition improved 1.7-fold from 6.7 to 11.5 d. This parameter allows an assessment of the availability of energy reserves in flies after mating and deposition of eggs and indicates to what extent adult feeding affects their metabolic maintenance. Given that most females die a few hours to four days postoviposition, the 5% honey treatment resulted in an exceptional longevity [15,34]. Samayoa et al. postulated a maximum longevity of 9 d postoviposition, while we observed individual females living up to 22 d [35]. This proves that the BSF adults are not only able to digest honey but can also metabolize it into energy that improves the aspects of their fitness. Nonetheless, it is economically advisable to establish a productive period in which flies mate and lay eggs rather than occupying cages for their entire adult lifespans. The cages can then be stocked with newly emerged flies to avoid increasing costs due to space occupancy. These results are an extension of the work of Romano et al. [45]. These authors conducted a sugar preference assay and noticed that when presented with white sugar, brown sugar, honey and water on different Petri dishes, the number of adult BSF that chose to feed on honey (55%) was significantly higher than any other diet (around 15% for brown sugar, 16% for white sugar, and 14% for water). While this study provided preference information, it did not provide data on how honey affects oviposition and longevity. Additionally, the sex ratio of the population was not determined, and only those flies that were observed on one of the Petri dishes within five days were included in the evaluation. As a result, multiple counting cannot be ruled out [45].

The fact that some studies have estimated a number of 500–1000 eggs per female [46] while others have estimated the number to be in the range of 205–820 [47] might suggest that the quantity of eggs laid by females is influenced by external factors, such as the availability of feed [12]. Tomberlin et al. [10] hypothesized that females may reabsorb oocytes in order to metabolize them to maintain respiration, as a result of which the egg clutch size would be reduced. Providing BSF with a rich diet that fulfils their energetic needs might thus not only increase the egg clutch size but also encourage females to stop reabsorbing oocytes after the first oviposition. This could result in the maturation of more oocytes and laying eggs multiple times (see 5% honey) instead of the one-time oviposition commonly described for BSF. Nevertheless, this does not necessarily suggest that all carbohydrates improve BSF adults’ rearing. As a matter of fact, even though honey itself contains glucose, a supplementation with pure glucose did not improve the number of eggs laid nor the longevity of the flies (Table 4). The same pattern was observed in the study of Romano et al., as no general preference was determined between water and brown or white sugar (mainly disaccharide sucrose) provided to flies on Petri dishes (all 14–16%) [45]. On the other hand, it was demonstrated that a sucrose solution (1:1 volume) significantly improved longevity, as males lived on average 32.7 d after emergence, while females lived 20.9 d compared to 19.7 d and 19.9 d, respectively, when fed with tap water [41]. Furthermore, the authors used a single-specimen approach, meaning that females were separated from males and did not mate or lay any eggs, affecting their longevity and making an accurate comparison with our data difficult. Honey is a natural resource made by bees from the nectar of flowers, the chemical composition of which depends on geography. Apart from small amounts of sucrose and more than 22 other sugars, honey mainly consists of the monosaccharides fructose (38.2%) and glucose (31.3%) [48]. However, the complex mixture contains a large number of other organic and inorganic compounds such as proteins and amino acids, organic acids, minerals, phenols, pigments, vitamins, and a variety of trace elements [49], which could also have a positive effect on the life history traits of BSF adults. This mixture could be more beneficial for adult BSF than individually purified compounds.

However, even though honey gave the best results compared to other treatments, a high concentration of 50% was lethal, opposed to other species, such as the aphid parasitoid *Aphidius ervi* (Hymenoptera: Braconidae). For this particular species, longevity increased significantly with increasing honey concentration and reached a maximum at 50–70% (males) or 70% (females) in the solution [50]. In contrast, BSF adults provided with 5–10% solutions not only lived longer but also achieved a significantly higher fecundity and egg yield than the 25% and 50% solutions, proving that lower doses are more suitable for BSF adult feeding. Such a positive association between longevity and fecundity in honey-fed BSF represents a reversal of the general fecundity–longevity trade-off described for some insect species [51]. In particular, positive associations were also found in eusocial insects such as *Bombus terrestris* (Hymenoptera: Apidae) and *Platythyrea punctata* (Hymenoptera: Formicidae) [52,53]. The extent to which female longevity affects fertility and hatchability rates should be examined in future studies.

Once the water evaporated, especially at the high carbohydrate concentrations (honey and glucose), we observed that concentrated substrate remains in the box and the flies can stick to it, particularly by their wings. As they fought to escape from the concentrated substrate, their wings were sometimes damaged or lost in the process, and in all these boxes, the females did not succeed at laying eggs even though they were alive long enough. In addition, the energy required for liberation could be the reason why the flies die earlier with increasing carbohydrate concentration. This could also be an explanation for the decrease in the oviposition period at high honey concentrations. Moreover, it confirms the importance of wing fanning during courtship prior to the males’ mounting attempts and is consistent with a study discussing the role played by wing fanning in evoking the behavioral responses of males [54].

In contrast to the carbohydrate-rich diets, preliminary feeding experiments with both protein powders revealed a high lethality when supplemented with 5%. Consequently, they were supplemented with 0.05–0.5%. For supplementation with proteins from cyanobacteria (*Spirulina*) and microalgae (*Chlorella*), the results obtained did not show any significant improvement in the reproductive parameters or the longevity of the flies compared to tap water (Table 5, Table 6). This is consistent with the findings of Lupi et al. [41], who used a protein source (meat broth) and noticed that even though the longevity was higher than in the starvation treatment, it was similar to water, as males lived less than 1.5 d and females 0.5 d longer. Contrarily, our data suggest that adult longevity is negatively affected by both cyanobacterial and microalgal supplements, as they have caused the flies to die 3.6–5.5 d earlier when fed at the lowest concentration (0.05%).

This might be because microalgae and cyanobacteria contain, unlike animal protein, considerable amounts of chlorophyll [55]. There is currently no evidence on the capabilities of BSF adults to digest chlorophyll and whether the insects possess the appropriate digestive enzymes to do so. Additionally, it was reported that chlorophyll derivatives, such as chlorophyllin and pheophorbide, can be used as photosensitizers [56]. For instance, both substances were shown to induce photodynamic damage in larvae of the dipteran mosquito genera *Aedes*, *Anopheles*, and *Culex* (all Diptera: Culicidae) as well as in *Chaoborus crystallinus* (Diptera: Chaoboridae), a representative of the phantom midges [56,57,58]. Those derivatives are highly effective even in low doses. For *C. chrystallinus*, a dose of only 8 ng chlorophyllin per larva caused necrosis and apoptosis, resulting in larval death [59]. A further cause might be that a high protein content alone is not sufficient to achieve positive effects in BSF adults, as high protein consumption was previously suggested to limit the longevity in *Drosophila melanogaster* (Diptera: Drosophilidae) [60,61]. In general, algae are considered a valuable source of protein and are often compared to soybean or egg. However, the quality of the protein may not be optimal for the adult BSF since amino acids such as tryptophan and lysine are particularly limiting in many algae [62,63].

In reality, BSF might be capable of digesting higher doses of carbohydrates than proteins, which is the case for other flies like *D. melanogaster*. In fact, for these fruit flies, studies have reported that optimal longevity was achieved when they were fed a mixed diet of high-carbohydrate and low-protein content. Here, the authors concluded an optimal ratio of carbohydrates:proteins between 10:1 and 20:1 for males [61] and of 16:1 for females [64]. In addition, the diet was not only shown to influence the adult BSF longevity but to affect the digestive physiology at the mRNA level. Bruno et al. found higher levels of α-glucosidase mRNA in the flies fed with sugar compared to the starved ones [12]. Carbohydrates and proteins have in general the same gross energy—17 kJ/g (4 kcal/g). On the other hand, carbohydrates are fast-acting and turn into energy as soon as they are ingested, thus representing an immediate energy source for flying. For instance, the hawkmoth *Amphion floridensis* (Lepidoptera: Sphingidae) relied primarily on carbohydrates when fed, whereas unfed ones burned almost exclusively fat reserves [65]. Therefore, it can be assumed that proteins are more difficult to access for the adult BSF and that specific monosaccharides are a more favorable energy source. Several studies on insects like *D. melanogaster*, *Anastrepha ludens* (Diptera: Tephritidae), and *Bactrocera tryoni* (Diptera: Tephritidae) report increasing evidence that an optimal ratio of carbohydrates and proteins is a key determinant of longevity [61,64,66,67]. Consistent with these findings, Bertinetti and colleagues proved that a mixture of sugar, bacteriological peptone, and milk powder (ratio 3:1:1) fed to BSF adults increased the oviposition period by 10.2 d, the egg amount 2.8-fold, and the female longevity by 3.2 d compared to water [7]. These results suggest that BSF adults do benefit from a mixture of sugar and protein. Further research on how different carbohydrate:protein ratios affect the reproduction and longevity of BSF adults should be carried out. In the future, this could significantly improve the reproductive outcome, especially in an industrial rearing facility.

## Figures and Tables

**Table 1:** Composition, nutrition, and energy content of the carbohydrate- and protein-rich supplements according to the manufacturers’ declarations (- = not specified by the manufacturer).

	*Chlorella*	*Spirulina*	Honey	d-Glucose
Product name	Bio *Chlorella* powder	Bio *Spirulina* powder	Bio forest honey	d (+)-glucose monohydrate
Species	*Chlorella vulgaris*	*Arthrospira* sp.	(*Apis mellifera*)	-
Company	Narayana Verlag, Kandern, Germany	Piura, Dublin, Ireland	Alnatura, Darmstadt, Germany	Carl Roth, Karlsruhe, Germany
Protein (g /100 g)	66.0	67.0	0.4	-
Fat (g /100 g)	1.8	0.7	<0.1	-
Saturated fatty acids (g/100 g)	0.6	0.5	<0.1	-
Carbohydrates (g/100 g)	12.0	13.0	77.9	91.8
Fiber (g/100 g)	14.0	5.8	0.0	-
Gross energy (kcal/100 g)	340	339	313	343
Sodium chloride (g/100 g)	0.2	2.2	<0.1	-
Iron (mg/100 g)	76	53	-	-
Phosphorus (mg/100 g)	1100	-	-	-
Manganese (mg/100 g)	6	-	-	-
Zinc (mg/100 g)	2.6	-	-	-
Vitamin A (µg/100 g)	1440	1204	-	-
Vitamin B2 (mg/100 g)	0.4	-	-	-
Vitamin B6 (mg/100 g)	-	0.71	-	-
Vitamin B12 (µg/100 g)	60	-	-	-

**Table 2:** Life history traits and reproductive parameters of adult BSF that had access to different types of water (tap water, distilled water, 0.5% NaCl *w*/*v*) or no water (starvation). Data are means ± SD. Different letters (a–d) within a row indicate statistically significant differences between diets (*p* < 0.05; one-way ANOVA; Kaplan–Meier estimator for longevity-related data).

Parameters		*n*	Tap Water	Distilled Water	0.5% NaCl	Starvation
Adult preoviposition period (d)		*n* = 30	9.1 ± 1.1 ^a^	9.0 ± 0.7 ^a^	7.9 ± 0.4 ^a^	no eggs
Oviposition period (d)		*n* = 30	5.0 ± 0.8 ^a^	4.0 ± 2.2 ^a^	1.7 ± 0.9 ^a^	no eggs
Oviposition peak (d)		*n* = 30	8.3 ± 1.2 ^a^	9.0 ± 0.8 ^a^	7.8 ± 0.6 ^a^	no eggs
Oviposition span (min–max d)		*n* = 30	6–14	7–13	7–10	no eggs
Adult longevity (d)	♂	*n* = 30	20.5 ± 1.3 ^a^	19.0 ± 2.0 ^a^	10.7 ± 0.6 ^b^	6.4 ± 0.1 ^c^
	♀	*n* = 30	17.6 ± 1.0 ^a^	16.2 ± 1.3 ^ab^	12.9 ± 2.2 ^b^	6.3 ± 0.4 ^c^
	total	*n* = 60	19.1 ± 1.1 ^a^	17.6 ± 1.2 ^b^	11.8 ± 1.3 ^c^	6.4 ± 0.1 ^d^
Adult longevity (min–max d)	♂	*n* = 30	13–26	12–26	5–19	5–8
	♀	*n* = 30	10–28	9–26	7–21	5–8
	total	*n* = 60	10–28	9–26	5–21	5–8
AL50 (d)		*n* = 60	19.0 ± 2.8 ^a^	16.3 ± 0.9 ^ab^	11.3 ± 1.9 ^bc^	6.3 ± 0.5 ^c^
Female longevity after oviposition (d)		*n* = 30	6.7 ± 1.8 ^a^	6.1 ± 0.1 ^a^	4.8 ± 2.9 ^a^	no eggs
Fecundity (egg clutches/10 females)		*n* = 30	7.0 ± 0.8 ^a^	6.7 ± 1.2 ^a^	3.0 ± 0.0 ^b^	0.0 ± 0.0 ^c^
Egg clutch weight (mg)		*n* = 30	15.0 ± 2.1 ^a^	15.6 ± 0.2 ^a^	12.8 ± 2.0 ^a^	no eggs
Span of egg clutch weight (min–max mg)		*n* = 30	11.3–31.8	12.4–20.1	7.9–18.2	no eggs
Mean egg yield (mg eggs/10 females)		*n* = 30	106.9 ± 26.9 ^a^	104.0 ± 18.3 ^a^	38.5 ± 6.1 ^b^	0.0 ± 0.0 ^b^
Total egg yield (mg eggs)		*n* = 30	320.8	312.0	115.4	0.0 ± 0.0
Egg clutch size (eggs/clutch)		*n* = 30	626.5 ± 86.1 ^a^	651.4 ± 9.5 ^a^	534.3 ± 84.7 ^a^	no eggs
Span of egg clutch size (min–max eggs)		*n* = 30	471–1325	517–838	329–758	no eggs
Multiple oviposition events (yes/no (number))		*n* = 30	yes (2)	yes (1)	no	no
No oviposition (yes/no (number))		*n* = 30	yes (9)	yes (10)	yes (21)	yes (30)

**Table 3:** Life history traits and reproductive parameters of adult BSF that were fed with carbohydrate-rich honey solutions (5–50% *w*/*v*). Tap water served as reference. Longevity was determined for males (♂) and females (♀). Data are means ± SD. Different letters (a–e) within a row indicate statistically significant differences between diets (*p* < 0.05; one-way ANOVA; Kaplan–Meier estimator for longevity-related data).

Parameters		*n*	Tap Water	5% Honey	10% Honey	25% Honey	50% Honey
Adult preoviposition period (d)		*n* = 30	9.1 ± 1.1 ^a^	9.2 ± 1.1 ^a^	9.0 ± 1.4 ^a^	8.4 ± 0.3 ^a^	8.0 ± 0.3 ^a^
Oviposition period (d)		*n* = 30	5.0 ± 0.8 ^a^	7.3 ± 3.4 ^a^	8.0 ± 3.6 ^a^	4.0 ± 0.8 ^a^	1.7 ± 0.5 ^a^
Oviposition peak (d)		*n* = 30	8.3 ± 1.2 ^a^	7.3 ± 0.5 ^a^	8.6 ± 1.7 ^a^	8.7 ± 0.5 ^a^	7.7 ± 0.5 ^a^
Oviposition span (min–max d)		*n* = 30	6–14	6–19	5–20	6–15	7–9
Adult longevity (d)	♂	*n* = 30	20.5 ± 1.3 ^a^	21.4 ± 1.2 ^ab^	18.9 ± 0.7 ^ac^	14.7 ± 0.9 ^d^	10.5 ± 0.8 ^e^
	♀	*n* = 30	17.6 ± 1.0 ^a^	20.8 ± 0.8 ^b^	17.6 ± 0.9 ^ac^	13.1 ± 0.2 ^d^	10.1 ± 0.2 ^e^
	total	*n* = 60	19.1 ± 1.1 ^a^	21.1 ± 1.0 ^b^	18.3 ± 0.7 ^ac^	13.9 ± 0.6 ^d^	10.3 ± 0.3 ^e^
Adult longevity (min–max d)	♂	*n* = 30	13–26	14–34	14–27	10–18	7–17
	♀	*n* = 30	10–28	13–33	13–25	9–17	7–15
	total	*n* = 60	10–28	13–34	13–27	9–18	7–17
AL50 (d)		*n* = 60	19.0 ± 2.8 ^a^	20.0 ± 1.4 ^a^	17.7 ± 0.5 ^ab^	14.3 ± 0.5 ^b^	9.3 ± 0.5 ^c^
Female longevity after oviposition (d)		*n* = 30	6.7 ± 1.8 ^a^	11.5 ± 0.6 ^b^	8.4 ± 2.2 ^ac^	4.8 ± 0.4 ^ad^	2.4 ± 0.4 ^e^
Fecundity (egg clutches/10 females)		*n* = 30	7.0 ± 0.8 ^a^	9.0 ± 0.8 ^ab^	9.3 ± 0.5 ^ab^	6.3 ± 0.9 ^ac^	4.0 ± 0.8 ^c^
Egg clutch weight (mg)		*n* = 30	15.0 ± 2.1 ^a^	20.3 ± 1.0 ^a^	18.0 ± 3.3 ^a^	15.2 ± 2.9 ^a^	12.7 ± 2.9 ^a^
Span of egg clutch weight (min–max mg)		*n* = 30	11.3–31.8	10.7–33.0	5.8–41.7	3.4–42.0	8.7–22.1
Mean egg yield (mg eggs/10 females)		*n* = 30	106.9 ± 26.9 ^a^	182.4 ± 10.7 ^b^	167.7 ± 27.8 ^ab^	94.6 ± 16.4 ^ac^	52.5 ± 22.3 ^ac^
Total egg yield (mg eggs)		*n* = 30	320.8	489.5	487.0	308.9	157.6
Egg clutch size (eggs/clutch)		*n* = 30	626.5 ± 86.1 ^a^	847.4 ± 41.0 ^a^	751.1 ± 136.7 ^a^	633.8 ± 122.2 ^a^	527.8 ± 122.8 ^a^
Span of egg clutch size (min–max eggs)		*n* = 30	471–1325	271–1375	242–1738	163–1750	363–921
Multiple oviposition events (yes/no (number))		*n* = 30	yes (2)	yes (15)	yes (9)	yes (6)	yes (1)
No oviposition (yes/no (number))		*n* = 30	yes (9)	yes (3)	yes (2)	yes (11)	yes (18)

**Table 4:** Life history traits and reproductive parameters of adult BSF that were fed with d-Glucose solutions (5–50% *w*/*v*). Tap water served as reference. Longevity was determined for males (♂) and females (♀). Data are means ± SD. Different letters (a–e) within a row indicate statistically significant differences between diets (*p* < 0.05; one-way ANOVA; Kaplan–Meier estimator for longevity-related data).

Parameters		*n*	Tap Water	5% Glucose	10% Glucose	25% Glucose	50% Glucose
Adult preoviposition period (d)		*n* = 30	9.1 ± 1.1 ^a^	7.9 ± 1.5 ^ab^	7.6 ± 0.8 ^ab^	6.4 ± 0.3 ^b^	6.1 ± 0.3 ^b^
Oviposition period (d)		*n* = 30	5.0 ± 0.8 ^a^	3.3 ± 1.9 ^a^	3.3 ± 1.2 ^a^	2.0 ± 0.8 ^a^	1.7 ± 0.5 ^a^
Oviposition peak (d)		*n* = 30	8.3 ± 1.2 ^a^	8.3 ± 1.2 ^a^	8.0 ± 1.4 ^a^	6.4 ± 0.3 ^a^	5.8 ± 0.6 ^a^
Oviposition span (min–max d)		*n* = 30	6–14	5–11	5–10	5–8	5–7
Adult longevity (d)	♂	*n* = 30	20.5 ± 1.3 ^a^	15.0 ± 0.6 ^b^	13.6 ± 0.4 ^c^	8.2 ± 0.5 ^d^	7.8 ± 0.4 ^d^
	♀	*n* = 30	17.6 ± 1.0 ^a^	13.6 ± 1.6 ^b^	13.1 ± 0.4 ^b^	8.0 ± 0.7 ^c^	8.0 ± 0.4 ^c^
	total	*n* = 60	19.1 ± 1.1 ^a^	14.3 ± 1.1 ^b^	13.3 ± 0.4 ^c^	8.1 ± 0.6 ^d^	7.9 ± 0.4 ^d^
Adult longevity (min–max d)	♂	*n* = 30	13–26	6–21	6–18	5–11	4–10
	♀	*n* = 30	10–28	6–23	5–18	5–12	4–10
	total	*n* = 60	10–28	6–23	5–18	5–12	4–10
AL50 (d)		*n* = 60	19.0 ± 2.8 ^a^	13.7 ± 1.7 ^b^	14.0 ± 0.0 ^b^	8.0 ± 0.8 ^c^	7.7 ± 0.5 ^c^
Female longevity after oviposition (d)		*n* = 30	6.7 ± 1.8 ^a^	5.6 ± 2.1 ^a^	6.1 ± 1.1 ^a^	2.0 ± 1.0 ^b^	2.1 ± 0.5 ^b^
Fecundity (egg clutches/10 females)		*n* = 30	7.0 ± 0.8 ^a^	5.7 ± 0.5 ^ab^	4.7 ± 0.5 ^bc^	3.0 ± 0.8 ^c^	2.7 ± 0.5 ^c^
Egg clutch weight (mg)		*n* = 30	15.0 ± 2.1 ^a^	14.2 ± 0.2 ^a^	15.2 ± 1.5 ^a^	12.3 ± 1.7 ^a^	11.8 ± 2.2 ^a^
Span of egg clutch weight (min–max mg)		*n* = 30	11.3–31.8	5.8–21.8	9.5–22.3	4.2–22.5	3.9–20.3
Mean egg yield (mg eggs/10 females)		*n* = 30	106.9 ± 26.9 ^a^	80.7 ± 6.3 ^ab^	71.7 ± 13.7 ^ab^	38.2 ± 14.4 ^b^	32.3 ± 10.6 ^b^
Total egg yield (mg eggs)		*n* = 30	320.8	241.0	215.1	114.6	96.9
Egg clutch size (eggs/clutch)		*n* = 30	626.5 ± 86.1 ^a^	593.4 ± 7.6 ^a^	633.8 ± 63.0 ^a^	512.8 ± 72.6 ^a^	490.0 ± 91.4 ^a^
Span of egg clutch size (min–max eggs)		*n* = 30	471–1325	242–908	396–929	175–938	163–846
Multiple oviposition events (yes/no (number))		*n* = 30	yes (2)	yes (4)	yes (2)	no	no
No oviposition (yes/no (number))		*n* = 30	yes (9)	yes (13)	yes (16)	yes (21)	yes (22)

**Table 5:** Life history traits and reproductive parameters of adult BSF that were fed with protein-rich *Spirulina* solutions (0.05–0.5% *w*/*v*). Tap water served as reference. Longevity was determined for males (♂) and females (♀). Data are means ± SD. Different letters (a–e) within a row indicate statistically significant differences between diets (*p* < 0.05; one-way ANOVA; Kaplan–Meier estimator for longevity-related data).

Parameters		*n*	Tap Water	0.05% *Spirulina*	0.1% *Spirulina*	0.25% *Spirulina*	0.5% *Spirulina*
Adult preoviposition period (d)		*n* = 30	9.1 ± 1.1 ^a^	7.7 ± 0.9 ^ab^	7.3 ± 0.5 ^ab^	6.3 ± 0.5 ^b^	5.7 ± 0.6 ^b^
Oviposition period (d)		*n* = 30	5.0 ± 0.8 ^a^	8.7 ± 1.7 ^b^	3.3 ± 0.9 ^a^	3.0 ± 0.8 ^a^	1.7 ± 0.5 ^a^
Oviposition peak (d)		*n* = 30	8.3 ± 1.2 ^a^	5.7 ± 1.2 ^a^	6.3 ± 0.5 ^a^	6.7 ± 0.5 ^a^	5.7 ± 0.5 ^a^
Oviposition span (min–max d)		*n* = 30	6–14	4–15	6–10	4–9	4–8
Adult longevity (d)	♂	*n* = 30	20.5 ± 1.3 ^a^	13.8 ± 1.3 ^b^	13.3 ± 1.5 ^b^	10.1 ± 0.8 ^c^	8.1 ± 0.5 ^d^
	♀	*n* = 30	17.6 ± 1.0 ^a^	13.4 ± 0.5 ^b^	13.9 ± 0.2 ^b^	9.6 ± 0.4 ^c^	7.6 ± 0.6 ^d^
	total	*n* = 60	19.1 ± 1.1 ^a^	13.6 ± 0.9 ^b^	13.6 ± 0.8 ^b^	9.9 ± 0.6 ^c^	7.9 ± 0.3 ^d^
Adult longevity (min–max d)	♂	*n* = 30	13–26	6–23	6–21	8–14	5–12
	♀	*n* = 30	10–28	9–17	11–18	7–13	5–13
	total	*n* = 60	10–28	6–23	6–21	7–14	5–13
AL50 (d)		*n* = 60	19.0 ± 2.8 ^a^	13.7 ± 0.5 ^b^	14.0 ± 0.8 ^b^	9.3 ± 0.5 ^c^	7.3 ± 0.5 ^c^
Female longevity after oviposition (d)		*n* = 30	6.7 ± 1.8 ^a^	5.5 ± 0.9 ^a^	6.4 ± 0.6 ^a^	3.4 ± 0.7 ^b^	1.7 ± 0.3 ^c^
Fecundity (egg clutches/ 10 females)		*n* = 30	7.0 ± 0.8 ^a^	7.0 ± 0.8 ^a^	6.0 ± 0.8 ^a^	6.7 ± 0.9 ^a^	5.0 ± 0.8 ^a^
Egg clutch weight (mg)		*n* = 30	15.0 ± 2.1 ^a^	15.8 ± 3.7 ^a^	15.0 ± 0.9 ^a^	16.2 ± 2.0 ^a^	15.5 ± 1.6 ^a^
Span of egg clutch weight (min–max mg)		*n* = 30	11.3–31.8	3.8–29.2	4.8–23.1	9.2–23.6	9.8–21.5
Mean egg yield (mg eggs/10 females)		*n* = 30	106.9 ± 26.9 ^a^	107.7 ± 13.4 ^a^	90.4 ± 16.4 ^a^	106.0 ± 0.5 ^a^	76.4 ± 6.6 ^a^
Total egg yield (mg eggs)		*n* = 30	320.8	323.1	271.1	318.1	229.2
Egg clutch size (eggs/clutch)		*n* = 30	626.5 ± 86.1 ^a^	659.0 ± 154.0 ^a^	623.6 ± 39.4 ^a^	674.7 ± 84.9 ^a^	646.5 ± 68.1 ^a^
Span of egg clutch size (min–max eggs)		*n* = 30	471–1325	158–1217	200–963	383–983	408–896
Multiple oviposition events (yes/no (number))		*n* = 30	yes (2)	yes (2)	no	no	yes (1)
No oviposition (yes/no (number))		*n* = 30	yes (9)	yes (9)	yes (12)	yes (10)	yes (15)

**Table 6:** Life history traits and reproductive parameters of adult BSF that were fed with protein-rich *Chlorella* solutions (0.05–0.5% *w*/*v*). Tap water served as reference. Longevity was determined for males (♂) and females (♀). Data are means ± SD. Different letters (a–e) within a row indicate statistically significant differences between diets (*p* < 0.05; one-way ANOVA; Kaplan–Meier estimator for longevity-related data).

Parameters		*n*	Tap Water	0.05% *Chlorella*	0.1% *Chlorella*	0.25% *Chlorella*	0.5% *Chlorella*
Adult preoviposition period (d)		*n* = 30	9.1 ± 1.1 ^a^	7.4 ± 0.9 ^ab^	6.4 ± 1.2 ^b^	5.7 ± 0.5 ^b^	6.0 ± 0.6 ^b^
Oviposition period (d)		*n* = 30	5.0 ± 0.8 ^a^	7.7 ± 0.5 ^a^	6.7 ± 3.3 ^a^	2.0 ± 0.8 ^a^	3.7 ± 2.4 ^a^
Oviposition peak (d)		*n* = 30	8.3 ± 1.2 ^a^	5.0 ± 1.4 ^a^	5.3 ± 0.5 ^a^	6.0 ± 0.8 ^a^	6.2 ± 0.3 ^a^
Oviposition span (min–max d)		*n* = 30	6–14	3–13	3–15	4–7	4–11
Adult longevity (d)	♂	*n* = 30	20.5 ± 1.3 ^a^	16.9 ± 1.2 ^b^	15.7 ± 0.1 ^b^	10.5 ± 0.3 ^c^	9.5 ± 0.2 ^d^
	♀	*n* = 30	17.6 ± 1.0 ^a^	14.1 ± 0.5 ^b^	13.7 ± 0.1 ^b^	10.5 ± 0.6 ^c^	9.6 ± 0.4 ^d^
	total	*n* = 60	19.1 ± 1.1 ^a^	15.5 ± 0.8 ^b^	14.7 ± 0.1 ^b^	10.5 ± 0.4 ^c^	9.6 ± 0.3 ^d^
Adult longevity (min–max d)	♂	*n* = 30	13–26	6–26	6–25	8–13	7–12
	♀	*n* = 30	10–28	9–19	11–16	8–13	7–12
	total	*n* = 60	10–28	6–26	6–25	8–13	7–12
AL50 (d)		*n* = 60	19.0 ± 2.8 ^a^	14.0 ± 0.0 ^b^	14.0 ± 0.0 ^b^	10.7 ± 0.5 ^bc^	9.3 ± 0.9 ^c^
Female longevity after oviposition (d)		*n* = 30	6.7 ± 1.8 ^a^	6.6 ± 0.3 ^a^	7.4 ± 1.5 ^a^	4.9 ± 1.3 ^b^	4.0 ± 0.9 ^b^
Fecundity (egg clutches/10 females)		*n* = 30	7.0 ± 0.8 ^a^	7.3 ± 0.5 ^a^	7.3 ± 0.9 ^a^	3.3 ± 1.2 ^b^	4.0 ± 0.8 ^b^
Egg clutch weight (mg)		*n* = 30	15.0 ± 2.1 ^a^	16.7 ± 0.8 ^a^	15.0 ± 0.7 ^a^	15.8 ± 1.9 ^a^	14.4 ± 2.4 ^a^
Span of egg clutch weight (min–max mg)		*n* = 30	11.3–31.8	5.1–24.5	6.2–27.3	9.2–20.2	7.9–21.3
Mean egg yield (mg eggs/10 females)		*n* = 30	106.9 ± 26.9 ^a^	122.5 ± 13.4 ^a^	109.0 ± 10.0 ^a^	50.3 ± 12.4 ^b^	55.8 ± 5.2 ^b^
Total egg yield (mg eggs)		*n* = 30	320.8	367.6	327.1	150.8	167.4
Egg clutch size (eggs/clutch)		*n* = 30	626.5 ± 86.1 ^a^	694.3 ± 32.3 ^a^	623.1 ± 31.1 ^a^	658.3 ± 80.2 ^a^	600.6 ± 101.4 ^a^
Span of egg clutch size (min–max eggs)		*n* = 30	471–1325	213–1021	258–1138	383–842	329–888
Multiple oviposition events (yes/no (number))		*n* = 30	yes (2)	yes (5)	yes (5)	no	yes (1)
No oviposition (yes/no (number))		*n* = 30	yes (9)	yes (8)	yes (8)	yes (20)	yes (18)

## Data Availability

All relevant data are contained within the article.
